# Seafood Discards: A Potent Source of Enzymes and Biomacromolecules With Nutritional and Nutraceutical Significance

**DOI:** 10.3389/fnut.2022.879929

**Published:** 2022-04-08

**Authors:** Moupriya Nag, Dibyajit Lahiri, Ankita Dey, Tanmay Sarkar, Siddhartha Pati, Sanket Joshi, Hamidun Bunawan, Arifullah Mohammed, Hisham Atan Edinur, Sreejita Ghosh, Rina Rani Ray

**Affiliations:** ^1^Department of Biotechnology, University of Engineering & Management, Kolkata, India; ^2^Department of Pathology, Belle Vue Clinic, Kolkata, India; ^3^Department of Food Processing Technology, Malda Polytechnic, West Bengal State Council of Technical Education, Government of West Bengal, Malda, India; ^4^Skills Innovation and Academic Network Institute, Association for Biodiversity Conservation and Research (ABC), Balasore, India; ^5^NatNov Bioscience Private Limited, Balasore, India; ^6^Central Analytical and Applied Research Unit, Oil & Gas Research Center, Sultan Qaboos University, Muscat, Oman; ^7^Institute of Systems Biology (INBIOSIS), Universiti Kebangsaan Malaysia, Bangi, Malaysia; ^8^Department of Agriculture Science, Faculty of Agro-Based Industry, Universiti Malaysia Kelantan Kampus Jeli, Jeli, Malaysia; ^9^School of Health Sciences, Health Campus, Universiti Sains Malaysia, Kubang Kerian, Malaysia; ^10^Department of Biotechnology, Maulana Abul Kalam Azad University of Technology, West Bengal, Kolkata, India

**Keywords:** sea food wastes, nutraceuticals, nutrients, enzymes, bioactive compounds, microbial conversion

## Abstract

In recent times, the seafood industry is found to produce large volumes of waste products comprising shrimp shells, fish bones, fins, skins, intestines, and carcasses, along with the voluminous quantity of wastewater effluents. These seafood industry effluents contain large quantities of lipids, amino acids, proteins, polyunsaturated fatty acids, minerals, and carotenoids mixed with the garbage. This debris not only causes a huge wastage of various nutrients but also roots in severe environmental contamination. Hence, the problem of such seafood industry run-offs needs to be immediately managed with a commercial outlook. Microbiological treatment may lead to the valorization of seafood wastes, the trove of several useful compounds into value-added materials like enzymes, such as lipase, protease, chitinase, hyaluronidase, phosphatase, etc., and organic compounds like bioactive peptides, collagen, gelatin, chitosan, and mineral-based nutraceuticals. Such bioconversion in combination with a bio-refinery strategy possesses the potential for environment-friendly and inexpensive management of discards generated from seafood, which can sustainably maintain the production of seafood. The compounds that are being produced may act as nutritional sources or as nutraceuticals, foods with medicinal value. Determining utilization of seafood discard not only reduces the obnoxious deposition of waste but adds economy in the production of food with nutritional and medicinal importance, and, thereby meets up the long-lasting global demand of making nutrients and nutraceuticals available at a nominal cost.

## Introduction

More than 70% of the earth’s surface is covered by water bodies comprising oceans. It has been seen that the biological diversity found within the tropical rain forests are found to be lower than those available within the marine environment. The marine system comprises enormous varieties of biodiversity that contain different types of bioactive compounds. Major groups of marine animals are soft-bodied, thereby requiring various means of defense systems by the production of various types of biochemical compounds that act as toxic substances for other animals (1, 2). The marine biodiversity, comprising various microflora, acts as an important biotherapeutic agent. The biotherapeutic compounds are the group of secondary metabolites being produced by microflora and fauna (3). The seafood comprises various types of organisms that are obtained from freshwater, estuarine, and brackish habitats (4).

In a larger sense, seafood includes shellfish and finfish from estuarine, marine, freshwater, and brackish ecosystems, and accounts for a significant portion of global production of food. In accordance with the Food and Agriculture Organization (FAO) State of World Fisheries and Aquaculture report, 167.2 million metric tons (MMT) of seafood were available worldwide in 2014, providing around 20 kg fish per capita (3). Mackerel, anchovies, herring, tuna, whiting, cod, and other species of finfish are common, whereas shellfish are divided into two groups based on structural characteristics: mollusks and crustaceans. Crustaceans include lobster, shrimp, crab, crayfish, and krill, whereas mollusks include cephalopods (cuttlefish, squid, and octopus), bivalves (oysters, mussels, scallops, and clams), and gastropods (sea snail, abalone, whelks, and cockle). In 2014, aquaculture produced 73.8 MMT favored seafood items, including 16.1 MMT mollusks, 49.8 MMT finfish, and 6.9 MMT crustaceans, owing to rising consumer needs (3). Bycatch from commercial fish processing frequently produces vast quantities of bycatch with little consumer appeal, which is typically disposed of as waste, animal feeds, or fertilizer. Marketable processing of seafood results in a massive number of discards, which can account for up to 50% of overall landings and include head, shell, guts, bones, skin, fin, and other items (5). Essential amino acids, peptides, carotenoids, long-chain omega-3 polyunsaturated fatty acids (PUFAs), vitamins B_3_, A, B_12_, B_6_, and D; and a variety of minerals for example copper, calcium, sodium, zinc, selenium, potassium, pigments, iodine and others are all abundant in bycatch, seafood of high value and processing wastes (6). Furthermore, they contain a variety of nutraceuticals, including lipid, nitrogen, mineral, and polysaccharide-based nutraceuticals. Proteins, such as gelatin and collagen; protein hydrolysates; peptides with remarkable biological activities; lipids enriched with long-chain polyunsaturated fatty acids (PUFA); hydrocarbons like carotenoids; squalene; polysaccharides like chitosan, chitin, glycosaminoglycans, and derivatives; and mineral-based products like bone powders, are examples of these compounds. These, based on their nature, possess interesting biological activities that could lead to the fabrication of functional foods and applications, such as organic food supplements, medicines, encapsulation, and nutraceutical carrier materials (6–9). With shifting customer preferences for natural bioactive ingredients for speedy scientific advancements and healthcare, there is a lot of room for secondary seafood processing and processing wastes for nutraceutical separation. This effort also aids in the entire utilization of the food for human consumption, hence, reducing seafood-related environmental issues. This article examines the nutraceuticals, nutrients, and other biomacromolecules found in edible fish pieces, as well as processing wastes of seafood. A brief introduction to functional foods and nutraceuticals is offered at the outset.

## Nutraceuticals

The word nutraceutical was coined from two words, nutrition and pharmaceutical, by Stephen DeFelice, MD, and chairman and founder of the Foundation for Innovation in Medicine (FIM) in Cranford, New Jersey in the year 1989 (10). DeFelice described nutraceutical as any portion of a food or a food providing health or medical benefits including treatment and/or prevention of a disorder. When functional food helps in treating and/or preventing disorder (s) and/or disease (s) except anemia, then that functional food is termed as a nutraceutical. It must be known that the word nutraceutical, as generally utilized for selling, does not have any regulatory explanation. Hence, nutraceuticals are not the same as dietary supplements because of the following reasons:

(i) Nutraceuticals can be used as traditional foods or as a solo item of a diet or a meal.
(ii) Nutraceuticals not only just supplement the diet but also help in treating and/or preventing disorder and/or disease.

Dietary constituents play a major beneficial role apart from providing basic nutrition, resulting in the designing of the concepts of nutraceuticals and functional foods (11). A functional food from one consumer may serve as a nutraceutical for another consumer. Nutraceutical examples comprise citrus fruits (such as orange juices) or tropical fruits (like mango and pineapple) (12), and fortified dairy products (13).

Numerous naturally produced food components have been used for cancer therapeutic strategies. Selenium, Vitamin D, Vitamin E, soy, green tea, and lycopene are those nutraceuticals, which have been extensively studied for human health benefits (10). Although most of these natural substances have been observed to possess immensely high potential for therapies, future investigations must involve well-fabricated clinical trials for assessing the synergistic actions of these substances, so that they can be used in human therapies.

The PUFAs including omega-6 and omega-3 fatty acids, and some phytochemicals even play a significant function in enhancing the health benefits of these bioactive dietary compounds. A balanced constitution of PUFA in foods impacts the divergent aspects of metabolism and immunity (11). Furthermore, interactions among portions of the gut microbiota with that of PUFA can also impact their bioactivities.

Biologically active non-nutritious plant compounds, also known as phytochemicals, are interesting for human nutrition due to their potential activities as antiestrogenic, antioxidants, immunomodulatory, anti-inflammatory, and anticarcinogenic (14). For instance, gut microbiota can impact and transform polyphenolic activities and their bioavailability (11). Phytochemicals along with their metabolic by-products can also prevent pathogenic bacterial growth while promoting the growth of beneficial bacteria, exerting effects like that of prebiotics. Interactions among functional components of food such as probiotics, prebiotics, and phytochemicals with that of the microbiota of the intestines have impacts on human health.

In broad terms, nutraceuticals play a major role in human beings *via* the maintenance of their normal wellbeing and physiological functions. While fish has been always dominant and superior over other proteinaceous functional foods or nutraceuticals, research-based studies on functional foods and nutraceuticals have already attained a saturation limit. Also, the awareness associated with the benefits of functional foods and nutraceuticals is lacking and must be underpinned (15). Awareness campaigns only can find reach within urban communities of countries and in some ways, the rural communities are neglected. Furthermore, based on a search result in PubMed by the use of keywords such as functional foods and nutraceuticals, many different research studies have been published with an increased pace from the year 1995.

### Fish Nutraceuticals

Fish nutraceuticals are used for improving health in different ways such as delaying the process of aging, preventing chronic and acute diseases, increasing expectancy of life, and supporting the fundamental functions and structure of the body (16). Population urbanization and awareness regarding health in people having either a stressed or sedentary lifestyle are the major reasons promoting the growth of the market for fish nutraceuticals all over the world (17). It has been recently reported that fish nutraceuticals can provide some positive strategies for managing human health exerting significant beneficial impacts on healthcare (18). A broad variety of phytochemicals like glucosinolates, terpenoids, limonoids, phytosterols, polyphenols, anthocyanidin, carotenoids, phytoestrogens, flavonoids, and isoflavonoids have imposed several therapeutic actions on human health including antioxidant, anti-inflammatory, anti-allergic, and antibacterial impacts (19). Such nutraceuticals, either alone or in synergy with other therapeutic strategies, not only aid in the maintenance of health by promotion of life quality, but also, prevent serious medical issues of the present time like cancer, diabetes, cholesterol, cardiovascular diseases, obesity, arthritis, and osteoporosis (20). Hence, cheap fish nutraceuticals are always exceptions in demand, especially among decreased income or economically susceptible groups. In this way, fish by-products or fishes help in solving the problem of malnutrition worldwide and other associated problems by providing important macro and micronutrients, easily biodegradable proteins, and high-density fats (21). All these important nutrients have several beneficial physiological functions in comparison to other proteins (22).

## Functional Foods

**FIGURE 1:**
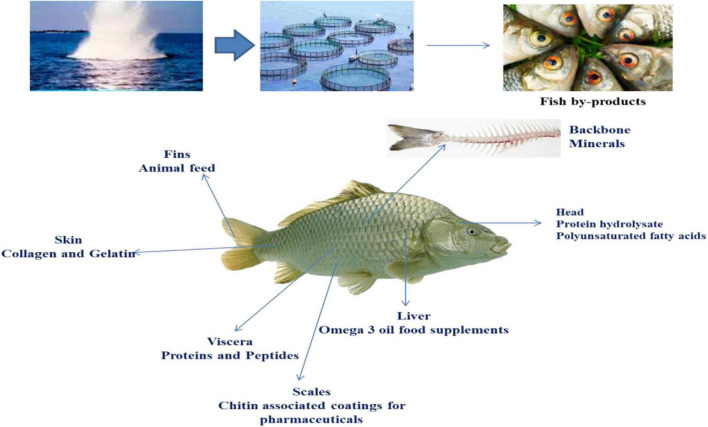
Fish and its by-products as nutraceuticals.

Functional foods are identical in terms of form to the usual foods; the functional foods being eaten as a component of the daily diet. On contrary to conventional foods, functional foods, on the other hand, have reduced risks of developing chronic diseases and exerted physiological benefits apart from basic nutritional benefits, including gut health maintenance (23). When food is being prepared or cooked using scientific intellect, without or with information regarding why and how it is being utilized, the food is termed as a functional food. Hence, functional foods supply the body with the necessary quantity of fats, vitamins, carbohydrates, and proteins required for its survival in healthy conditions (Figure 1).

## Discards of Seafood Processing

The food processing industries produce a large amount of waste that comprises both solid and liquid discards. The wastewaters that are released from these industries comprise portions of processed food that are released as effluents. It has been observed that globally, 1.3 million tons of waste are produced (5). Even though a huge number of species are harvested for seafood, only small portions are used and the remaining get wasted due to smaller size, unattractive colors, and high content of fat (5). Various types of food are produced from the marine system, 80% of them comprise smoked, dried, frozen, chilled, marinated, or fermented products (6).

About 80% of the overall harvest is processed into frozen, chilled, dried, smoked, marinated, or fermented products in the seafood business. De- shelling, beheading, gutting, skinning, removal of scales and fins, washing, fileting and other centralized pre-processing activities produce substantial quantities of solid discards and wastewater as effluents. Based on wet weight, the discards contain up to 50% entire shellfish including krill, shrimp, and crab. About 70% of shrimp discards are made up of the head and 30% is made up of the shell (6). The Argentine red shrimp (ARS) is a popular mollusk, whose industrialized processing produces 18,000 MT shell debris each year, causing ecological disturbances and pollution in Argentina’s Patagonia (9). Processing of lobster produces 50–70% shellfish as by-products, including shells, heads, eggs, and livers, totaling greater than 50,000 MT every year (8). Up to 80,000 MT shellfish discards are produced in India (7). Finfish waste accounts for 25–50% of raw materials and includes heads, entrails, skin, skeletal frames, viscera, and scales. Freshwater fishes, including carp, trout, bream, and pike, are processed to generate 40–60% fish discards (24). According to researchers, about half of the edible supply of seafood in the United States was unavailable for consumption by humans between 2009 and 2013 (25). This included bycatch, which accounted for 16–32% of the yield. In Europe, a nearly equal proportion of seafood is predicted to be thrown as trash for every ton being consumed. Each year, crab and shrimp processing in the EU generates larger than 100,000 MT shell trash (26). Furthermore, huge volumes of fresh biomass of fish will be produced in European ports as a result of the EU’s Landing Obligation Guidelines (27). Apart from solid waste, the seafood sector produces large volumes of wastewater as effluents due to processing operations like chilling, washing, fileting, blanching, marination, cooking, and others. According to calculations, every ton of raw seafood needs between 10 and 40 m^3^ of water (28). With a yearly production of around 50,000 MT, one of Europe’s largest processing plants for herring produces roughly 1,500 m^3^ of effluents every day (29). Production of surimi utilizes more quantity of water, as compared to curing, canning, or freezing since it entails frequent washing of fish minces (30). Water needs for each metric ton of fish in farmed production range between 1.5 and 6 m^3^ in the case of generic fish (31).

### Useful Seafood Discards

Seafood wastes can be utilized as raw materials for fish meal, silage, components of poultry and aquatic feed, and as a fertilizer primarily because of the high quantity of PUFA, proteins, and other nutrients such as minerals, carotenoids, squalene, vitamins, and glycosaminoglycans possessing different health benefits. Because of this factor, despite the low value conventionally assigned to the by-products of the fishery, there is ever-increasing attention toward the various utilizations of such discards as functional components, nutraceuticals, and pharmaceuticals with a broad spectrum of uses. On one hand, this strategy provides an important beneficial impact from a financial perspective, thereby providing an extra source of income from a substance, which in some instances may incur a cost of disposal. On the other hand, the valorization of seafood discards involving both by-products, as well as bycatch strongly decreases environmental pollution. Since aquaculture and fishery discards are enriched with high nutritional quality, there exists a great potential in the marine bioprocessing industries in converting and using a huge amount of these valuable wastes. For example, seafood consists of high quantities of proteins differing in biological and functional characteristics and this protein is particularly available at an increased concentration within the fish backbones, heads, and tails (8). Such proteins, along with some other biological molecules derived from seafood, particularly from shellfish, can be easily obtained by the use of new techniques designed for the biotechnological purpose just as in the instance of chitosan generated by exoskeleton from the shrimp *Pleoticusmuelleri* (32). These technological advances comprise biotransformation of biomolecules through microbes or enzymes, supercritical, or subcritical extraction for isolating target products, microwave, ultrafiltration, and ultrasound-assisted membrane separation and recovery procedures. Due to these reasons, energy-efficient and cheap enzymatic technologies are evolving in food processing industries based on the uses of glycoside hydrolases, proteases, transglutaminases, and lipases.

The pre-processing technologies of seafood from aquaculture and fisheries produce diverse waste products according to the type of raw material and the preferred finished products in various markets. Wastes generated from seafood processing involve de-shelling, beheading, gutting, skinning, scale and fin removal, washing, and fileting. This waste comprises 40% of the entire seafood. These materials are thrown off as solid wastes, by-products, or offal. The waste material percent may change depending on the organism being processed, like for example, in finfish, which produces about 50% waste products that include heads, entrails, skin, skeletal frames, viscera, and scales. Similar wastes are generated during the canning processes executed for tuna fish resulting in the production of 70% wastes.

Crustacean byproducts and wastes may reach up to 75% of the shellfish just like in processing industries of lobsters, which are composed of carapace, cephalothorax, shell, and tail. Wastes generated from shellfish are completely insoluble and extremely resistant to natural biodegradation, thereby leading to environmental and health concerns. However, the shell constituents usually consist of 30% chitin and 30% protein, which make them fascinating to be processed further. The main limitation of the valorization of biowastes from shrimps is the low shelf-life in a tropical climate and is rapidly degraded through bacterial actions. Different techniques have been, so far, designed to use wastes generated from shrimp replacing hazardous and standard chemical processes for biologically active compound extraction. Shellfish are enriched with carotenoids, which are lipophiles producing naturally red and yellow colors, and, particularly astaxanthin, is commercially used because of its role as an antioxidant and for aquacultures, where it is used as additives in feed to enhance the coloration of the flesh (pink color) of salmonids in farms, usually consumed by customers (33).

In addition to crustaceans and fishes, wastes produced from marine algae can also be exploited. In fact, from a nutritional perspective, edible seaweeds are enriched with vitamins and minerals, identified as an ideal iodine source, and one of the very few plant sources producing vitamin B12 (34, 35). Different types of seaweeds have been exploited traditionally for consumption by humans; Rhodophyta (*Porphyra*), Chlorophyta (*Ulva*), *Laminaria*, *Undaria*, Phaeophyceae (*Saccharina*), and *Himanthalia* constitute the usual ingredients in many Asian foods, thereby enhancing various food product quality. Wastes generated from macroalgae are not enough for satisfying the demand in the entire world and many species of algae are extensively cultivated particularly in integrated aquacultures.

### Biomacromolecules and Bioactive Compounds From Fish and By-Products of Fisheries

The term “bioactive” has been obtained from Greek and Latin words “life” and “dynamic,” meaning full of energy or participating in a role. Fishes, which are matchless fusions of bioactive compounds, such as omega-3-PUFA, long-chain PUFAs [decosahexanoic acid (DHA) and eicosapentaenoic acid (EPA)], protein hydrosylates, peptides, minerals, amino acids, gelatin, vitamins, fish oil, collagen, fat-soluble vitamins, and fish bones, make them a significant nutraceutical source. Because of the remarkable beneficial value of by-products from fisheries and of fish, several nutraceuticals are being used for preventive purposes in different medicinal fields. They can also be used for reducing the severity or for treating different diseases like cancer, cardiovascular disorders, rickets, viral diseases, hypertension, dermatological problems (particularly during pregnancy), and other helminthic infections (36, 37). Besides all such benefits, fish-based nutraceuticals also have anti-inflammatory, anti-coagulant, and antioxidant properties.

Usually, a huge quantity of fish by-products is either thrown off or utilized as low-value products. It has been found that approximately 25% of fish discards released by fisheries are disposed of, thus, causing a substantial effect on the environment, as well as potential loss of significant bioactive compounds present in fish wastes. Fish waste can be described as any species of fish or fish having almost zero or very low commercial significance of fishes that are caught in very few quantities not possessing many warrants on sale.

Furthermore, about half of the body parts of fishes, such as fins, heads, and viscera, as well as skin, are regarded as wastes in fisheries. Based on previous reports, every year, fisheries release over 20 million tons of fish wastes as a consequence of catching non-desired fishes or processing the fish wastes as by-products (36). These wastes comprise a large quantity of the total production of fish. According to the reports of the European Union, these fish discards amount to 5.2 million tons every year. Three main technologies (physical or chemical, microbial, and enzymatic methods) are used for the recovery of the bioactive compounds from these discards. Out of these three technologies, the enzymatic process is regarded to be one of the best technologies for all types of fish discards (fish skin, head, frames, viscera, and scales). Subsequently, it is necessary to identify the value of these fish discards that can be used for developing innovative functional foods and nutraceuticals from them. Thus, increased research needs to be conducted on fish nutraceuticals. Potently low-valued nutraceuticals can replenish the worldwide demand for dietary supplements, particularly for least developed or underdeveloped countries. Table 1 below describes some of the bioactive compounds from fish discards, which can be used in nutraceuticals having potential health benefits.

**TABLE 1:** Biomacromolecules from various fish discards and their potential health benefits.

Biomacromolecules	Health benefits	Fish	References
Omega- 6- fatty acids	Decreased risk of cardiovascular disorders, amelioration of diseases like hypertension and arthritis, oxidation, enhanced expression of vascular adhesion molecule- 1, vasoconstriction, aggregation of platelets, eicosanoid biosynthesis	Sardine, aquatic char, anchovies, fried calamari	(38, 39)
Omega- 3- fatty acids	Cardio-protective and anti-inflammatory effects, neuro and vision development, various types of cancers (colorectal, breast and prostate), inflammatory bowel disease (IBD), asthma, osteoporosis, rheumatoid arthritis, increases sensitivity of insulin	Spiny dogfish, mackerel, salmon, black halibut, sardines	(40)
Vitamin D	Osteomalacia, rickets, increased density of bones	*Sardinella longiceps*, *Amblypharyngodon mola*	(41)
Vitamin B complex	Converts food to energy within body cells and aids the nerve tissues to function properly	Shellfish, black sea fish	(42)
Vitamin A	Promotes growth, reduces poor vision, aids in growth of bones	*Amblypharyngodon mola*	(43)
Histidine	Precursors of various hormones (thyrotropin- release hormone), major metabolite for renal activities, neurotransmission, immune system and gastric secretion, anti-inflammatory and antioxidant properties, critical for metabolism and regulation of trace elements and precursor for histamine	*Catlacatla*, *Rastrelligerkanagurta*	(44)
Arginine	Essential to detoxify ammonia, essential nutrient for spermatogenesis, survival of embryo, neonatal and fetal growth, maintenance of hemodynamics and vascular tone	*Oncorhynchus mykiss*	(45)
Lysine	Required for optimum growth, serves as immunomodulator, treat, and prevent cold sores	*Rastrelligerkanagurta*, *Stolephoruswaitei*, *Stolephorus. commersonii*	(43)
Isoleucine	Aid in formation of muscle and appropriate growth	*Labeorohita*, *Oncorhynchus mykiss*	(43)
Phenylalanine	Tyrosine precursor	*Catlacatla*, *Cirrhinusmrigala*	(46)
Methionine	Used at multiple levels for metabolism in cells, as protein component, initiation of translation of mRNA, regulator molecule as S- adenosylmethionine	*Tor putitora*, *Stolephoruswaitei*	(47)
Tyrosine	Precursor of norepinephrine and dopamine	*Tor putitora*, *Oncorhynchus mykiss*	(48)
Threonine	Plays a major function in maintaining mucosal integrity in intestines and barrier function	*Nemipterusjaponicas*, *Thunnus albacores*	(49)
Glutamine	Substrate for biosynthesis of nucleotides (pyrimidines, purines, amino sugars), NADPH, antioxidants, other biosynthetic processes for maintaining cellular function and integrity	*Catlacatla*, *Labeorohita*, *Cirrhinusmrigala*	(50)
Valine	Protein biosynthesis, homeostasis of glucose, anti-obesity, nutritive- sensitive signaling processes	*Cirrhinusmrigala*, *Nemipterus japonicas*	(51)
Proline	Major function in cell differentiation in fetus, helps in extra- embryonic membrane development	*Tor putitora*, *Oncorhynchus mykiss*	(45)
Glycine	Gene expression regulation, activity and configuration of protein, numerous biological activities such as biosynthesis of glutathione	*Labeorohita*, *Catlacatla*, *Cirrhinusmrigala*	(52, 53)
Aspartic acid	Treats chronic fatigue by generating cellular energy	*Labeoniloticus*	(54)
Alanine	Protein biosynthesis acts as a remarkable source of carbon for hepatic gluconeogenesis	*Polypedates maculates*	(54)
Leucine	Promotion of energy metabolism (biogenesis of mitochondria, uptake of glucose and oxidation of fatty acid), for providing energy for protein biosynthesis and prevention of degradation of protein	*Lethrinusharak*	(54)
Glutamic acid	Buffer, surfactant, chelating agent, flavor enrichment, agriculture, serve as fuel, immune functions	*Labeoniloticus*	(54)
Serine	Proliferation and differentiation, cellular homeostasis	*Mugil cephalus*	(54)
Zinc	Helps in development and growth, proper immune cell functioning, healthy skin, cell growth and cell division, carbohydrate breakdown, wound healing. Enhances sense of taste and smell	*Rita Rita*, *Sperataseenghala*	(43)
Iron	Hemoglobin biosynthesis in RBCs	*Rita rita*, *Sperataseenghala*	(43)
Iodine	Essential hormones to regulate body metabolism and in children, required for normal mental development and growth		(43)
Calcium	Needed for formation and mineralization of strong bones and normal muscular and nervous functions	*Gudusiachapra*, *Xenentodoncancila*	(43)
Beta- carotene, astaxanthin, lutein, and zeaxanthin	Prevents cancer, acts as an antioxidant, anti-atherogenic, prevents cardiovascular and neurological disorders, psoriasis, ophthalmology, used in cosmetics and preservatives	Red fishes, freshwater fish and other fish	(55)
Chitosan and chitin	Accelerator for wound healing, reduction of levels of cholesterol in blood, anti-aging and anti-ulcer agent, ophthalmology	Fish scales	(56)
Bioactive peptides	Anti-oxidant, anti-hypertensive, anti- proliferative, and antimicrobial activities	Fish by-products, fish protein, and muscle	(57)
Glucosamine	Osteoarthritis, anti- inflammatory, supplements in diet	Fish discards	(58)
Chondroitin	Supplements in diet, osteoarthritis	Fish discards	(58)
Collagen	Reduces hypertension, osteoarthritis, acts as antioxidant, in tissue engineering, anti- skin aging, and anti- hypertensive agent	Fish discards	(38)
Gelatin	Food industries, pharmaceutical industries, dairy product stabilization, microencapsulation of vitamins	Fish discards	(59)
Squalene	Antioxidant seafood-related protective functions, anti- fungal, antibacterial and anti-cancer agents	*Tincatinca*, *Scardiniuserthrophthalmus*	(60)
			

## Biomacromolecules From Seafood Discards Used in Nutraceuticals

### Gelatin and Collagen

**FIGURE 2:**
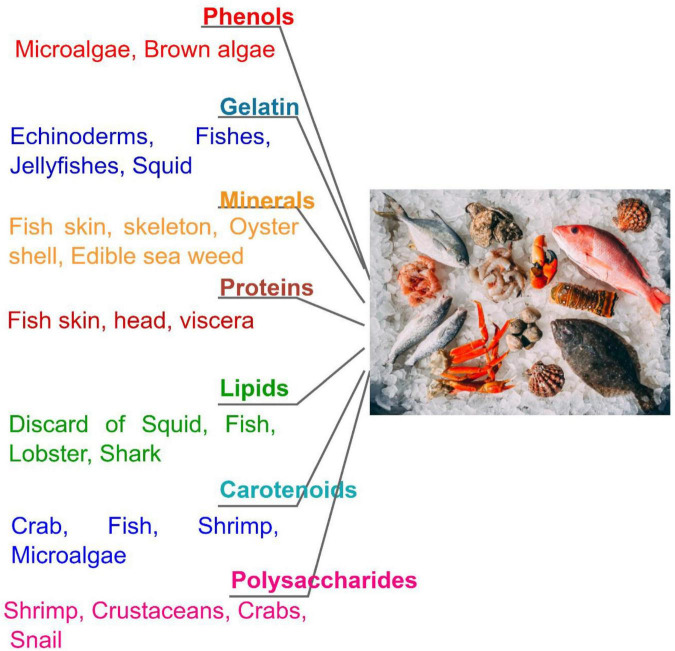
Biomacromolecules from seafood discards.

Collagen of types IV, II, and I are specifically derived from bones, skin, cartilages, and scales *via* sustainable strategies by the European zero wastes concept (61). Discards from echinoderms, fishes, and jellyfishes can be used as sources for collagen of good quality, which has been effectively isolated and being processed through alkaline, acidic, and enzymatic methods in combination with mechanical processes like adjustments of pH, sonication, and homogenization (Figure 2). Type I collagen is derived from the tissues of octopus, sea urchins, jellyfishes, starfishes, and numerous other fish species. The major amino acids obtained from gelatin and collagen molecules are alanine, glycine, hydroxyproline (Gly-X-Y repeating triplet unit), and proline. The composition of amino acids might vary based on environmental situations (like temperature), methods of extraction, and tissue types.

Over the recent years, collagen finds application in many health sectors mainly pharmaceutical and medical care (orthopedics, plastic surgery, dentistry, and ophthalmology). Apart from the increased potentials of gelatin and collagen in the development of new therapies and medicines, just a few instances of them being available as commercial drugs have been reported (62). In non-healthcare fields, a remarkable application of collagen is associated with the food industries (nutraceuticals and food processing), but gelatin is mostly used in these industries in its denatured state. Collagen has become a functional component in the development of healthy foods. Usually, the production of collagen reduces with an unhealthy diet and age, and hence, as a result, food supplements are needed for upholding the skin, nails, hair, and tissues of the human body. Gelatin from fishes can be extracted by denaturing and hydrolyzing collagen *via* a pre-treatment process required prior to its extraction, thereby enhancing the efficiency of extraction. The pre-treatment process comprises alkaline or acidic hydrolysis, a procedure selected based on source material and gelatin, which is prepared by water or acid extraction. Attributes of gelatin are impacted by two major factors: the process of extraction and the features of the source collagen material. Gelatin is broadly used as a component for improving texture, elasticity, and stability of food products and it may also lead to the extraction of bioactive peptides *via* hydrolysis with protease. Such metabolites possess a potential action as inhibitors of antioxidants or angiotensin I converting enzyme (ACE). For example, gelatin extracted by acid hydrolysis of four various species of local aquatic fishes, which are being caught off the coast of Langkawi Island, Malaysia, like *Rastrelliger kanagurta* (kembung), *Epinephelussex fasciatus* (kerapu), *Pristipomodes typus* (kerisi), and *Lutjianus argentimaculatus* (jenahak) consists of important amino acids with glycine as the most dominant one (63). Gelatin derived from tunics of giant squid (*Dosidicus gigas*) and tuna fishes showed antioxidant properties after being hydrolyzed with pepsin or α-chymotrypsin and trypsin (64). This study assured the presence of increased antioxidant potential of fractionated and whole alcalase gelatin hydrolysates of giant squid. This potential was remarkably high in comparison to that extracted from tuna fishes in presence of the identical conditions of hydrolysis.

In another study similar to this, the antioxidant effects of gelatin obtained from frozen outer and inner tunics of *Dosidicus gigas* (jumbo flying squid), *Thunnus* spp. (tuna), and *Hypoglossus* spp. (halibut) skins were tested by 2, 2′-azino-bis-ethylbenothiazoline-6-sulfonic acid (ABTS) and ferric reducing antioxidant power (FRAP) assays (65). Particularly, gelatin found from squid demonstrated a greater antioxidant effect because of iron reduction and enhancement of free radical elimination. Peptides extracted from gelatin of the skin in *Gadus macrocephalus* (Pacific cod), specifically papain hydrolysates, demonstrated potent antioxidant effect (66). Moreover, peptides derived by purifying papain hydrolysates demonstrated a potent inhibitory action of the enzyme ACE. The gelatin derived from *Oncorhynchus keta* (Chum salmon) skin and their hydrolysates possessed shielding activities against UV radiation-associated skin photoaging (67). Particularly, gelatin hydrolysates from salmon skins can be used for developing nutraceuticals for reduction of oxidative stress, maintenance of homeostasis of the matrix of collagen, and strengthening the system of immunity.

Ultrasound methods for collagen removal from the skins of *Lateolabraxjaponicas* (sea bass) have been fabricated by many researchers (68). Apart from this, the collagen obtained from edible giant jellyfish (*Nemopilemanomurai*) triggered cytokine and immunoglobulin production by the hybridoma cells of humans and human peripheral blood lymphocytes without stimulating allergic reactions (69).

### Minerals

Mineral compounds can be classified into micro minerals (trace minerals) like zinc, copper, iron, and manganese or macro minerals (major minerals) like sodium, calcium, and potassium.

Among these, calcium is the most basic element for vertebrate physiology for maintaining and building strong bones, and calcium is also required for other biochemical activities and tissue functions. Calcium obtained from food discards demonstrated good capacities of making bread enriched with calcium, and such bread can be utilized for treating patients suffering from calcium deficiencies and osteoporosis (17). It was observed that the skeletons were thrown off post-industrial polysaccharide-based *belengerii* (hoki) and its digested products can be utilized as nutraceuticals having potential calcium-binding capability (30). Milk tablets, which are supplemented with nanopowder oyster shell (NPOS) or nanopowder eggshell (NPES), NPOS having activated zinc (Zn- NPOS), and zinc NPES (Zn- NPES), are suitable for supplying calcium because it did not indicate any remarkable variations in consistency, pH, humidity, and color, in comparison to the control milk tablets (70). Furthermore, a bread nourished with oyster shells possessed ash, high protein, and fiber content than the control bread. Apart from this, oyster and eggshell bread had remarkably high levels of iron, calcium, phosphorous, and zinc as compared to the control. Bread fortification with natural calcium sources like skimmed milk powder or discards like eggshell or oyster shell powders was observed to enhance the rheological properties, nutritional attributes, and quality of the dough in comparison to the dough of the control bread (71). Even though some researchers studied the high nutritional quality of calcium from seafood discards as a food supplement, it should be taken into consideration that ionization and solubilization procedures are required for its actual adsorption. These issues create a barrier for using these aquatic calcium minerals for the consumption of humans (72).

Iron is a mineral that takes part in several biochemical processes like the production of energy, transportation of oxygen, and proliferation of cells and due to these reasons, iron is one of the most significant micro minerals in human physiology. On the contrary, almost one-fifth of the total population worldwide has some nutritional issues because of a deficiency of iron (73). Hence, iron can be provided *via* a diet rich in metal chelating substances, salt, and peptides that can chelate iron. For example, the Alaska pollock skin on hydrolysis with trypsin can generate peptides capable of chelating iron and are highly stable with adsorption characteristics necessary for serving as iron carriers in food supplements used in therapies (74).

Aquatic seaweeds comprise 10–100 times more quantity of minerals as compared to conventional vegetables with copper, iron, magnesium, and calcium being present at extremely high concentrations. Furthermore, seaweeds may be considered as the best economical food for fulfilling the necessities of iodine in humans, and usually, they can be utilized as minerals in food supplements and for their beneficial impacts on arterial hypertension and hypercholesterolemia. Because of their nutritional properties, algae are generally utilized in the form of dietary supplements, mainly algae of the genera *Chlorella* and *Spirulina* (75). For instance, *Laminaria japonica* is capable of storing aquatic minerals at high concentrations and it can be used for producing algae-based components to protect skin against damage due to UV (76).

### Proteins and Hydrosylates From Proteins

Although different molecules with anti-hypertensive properties are available in the market, however, typical side effects like angioedema and cough are very common according to clinical test data (77, 78). The synthetic inhibitors that are generally used in curative formulation, are to be replaced by a potential solution with organic peptides extracted from food proteins. Surprisingly, bio-active peptides from the marine ecosystem have numerous biological characteristics like inhibition of ACE, anticoagulant and antimicrobial properties, immune-modulatory properties, and antioxidant properties. Considering the nutritional point of view, the protein hydrolysates of fish, because of their composition of amino acids and proteins that are easily digestible, are considered to have the best quality. However, these are widely utilized for animal nutrition because of their uncomfortable smell and flavor. Present-day research has provided significant indications that bio-active peptides of the marine ecosystem can be used as antioxidants that inhibit lipid peroxidation and also help to eliminate reactive oxygen strains (79). Incidentally, the scavenging activities of free radicals are directed to the hydro-phobic amino acids like isoleucine, glycine, phenylalanine, tyrosine, cysteine, and others, which enhance the efficiency level of the antioxidant peptides. Those amino acids can be used as proton donors or lipid-radical scavengers. For example, when examined within a linoleic acid peroxidation arrangement and radical scavenging potency, peptides like His-Gly-Pro-Leu-Gly-Pro-Leu (797 Da) taken out from the skin gelatin of *Johnius belengerii* (Hoki) fish exhibit antioxidant properties. Adding to this, it also indicates that this is necessitated in the redox balance maintenance of the cell environment as these peptides provoke the increment of the levels of anti-oxidative enzymes inside cultured hepatoma cells of humans (80).

For getting a retorted skin gelatin hydrolysate (RSGH), the skin of *Rachycentron canadum* (Cobia), pre-treated with alkaline, is taken out in a retort after 30 min. This also indicated that RSGH from Cobia and its derivatives have a significant antioxidant property as it inhibits lipid peroxidation. It is a settled fact that the quality of food degrades and ends up in rancidity, improper taste, and reduced shelf life because of lipid peroxidation. The RSGH can be utilized as an organic antioxidant in food products. The functional foods and supplements can be improved by using those peptides as antioxidants (81). In a different study, the liposomes and DPPH (2,2-diphenyl-1-picrylhydrazyl) radical scavenging assay were utilized for examining the antioxidant property of protein hydrolysates extracted from the backbones of *Gadus morhua* (cod). This scavenging activity of the DPPH indicated that this anti-oxidative property of hydrolysates might be because of its capability to scavenge lipid radicals (82). Antioxidant activities were also found out in the protein hydrolysates that were obtained from various enzymatic therapies of sardine heads and viscera (*Sardinella aurita*), from the skin of Alaska pollack (*Theragrachalcogramma*), from sea bream (*Nemipterus japonicus*) muscles, and some other fishes like *Exocoetus volitans* (83). Furthermore, the protein hydrosylates from trypsin in all the fishes demonstrated the most free radical scavenging activity and inhibition of lipid peroxidation. Moreover, a strong anti-proliferative effect was found in these peptides on the human hepatocellular liver carcinoma (Hep G2) cell line (84). A peptide showed a typical antioxidant property when extracted from the viscera of *Parastromateusniger* (black pomfret), and could slow up both lipid peroxidation and oxidative damage (85). The protein hydrolysates from the viscera of the horse mackerel fish (*Magalaspis cordyla*) also showed the same activity. This peptide, without any oxidative damage to the living system, can inhibit lipid peroxidation (86). *In vitro* assay of two peptides exhibited antioxidant activities when extracted from the protein hydrolysates of the skin of fishes, horse mackerel (*Magalaspiscordyla*), croaker (Otolithes ruber), and the cuttlefish (*Sepia officinalis*) by-products, like viscera and skin, gave eight hydrolysates when treated with different gastrointestinal proteases like crude alkaline enzyme, trypsin, chymotrypsin, and proteases from bacteria (87).

In connection with the antioxidant properties, the hydrophobic characteristics of peptides, as well as modifications in their chain length and sequences in the amino acid, have been linked to ACE inhibition action (88). For example, it was found that an ACE inhibitor hydrophobic peptide Gly-Leu-Pro-Leu-Asn-Leu-Pro, with a molecular weight of 770 Da, isolated from salmon skin helps to decrease the systolic blood pressure in rats when orally administered, and this peptide had an anti-hypertensive activity and can be used as a peptide for functional food formulation (89). The jellyfish extracts from three Mediterranean species (*Cotylorhiza tuberculate*, *Aurelia* spp., and *Rhizostomapulmo*) of it have exhibited antioxidant properties having favorable use as nutraceuticals in food industries (90). It was found that antioxidant and ACE inhibitory molecules, Ser-Tyr dipeptide, abundantly found in the gonads from different species of jellyfishes, such as *Rhopilema esculentus* (Kishinouye), had hydroxyl, DPPH, and superoxide radical scavenging properties with IC50- 1177.63, 84.62, and 456.66 μM, respectively (91).

As indicated earlier, in the development of nutraceutical products, macroalgae can be utilized. Using the colorimetric method, two peptides, Glu-Leu-Ser, and Gly-Gly-Ser-Lys extracted from proteolytic enzymes hydrolysates of red seaweed laver (*Porphyra*sp.), stopped the α-amylase activity at 1 mg/ml. It was proposed that seaweed hydrolysates can be used in the treatments of diabetes as this enzymatic activity helps to decrease blood glucose levels (92). Another novel peptide from hydrolysate of Nori might be utilized as a functional food for avoiding thrombosis as it minimized the blood clotting factors that were involved in the innate pathway of coagulation (93).

### Lipids

Lipids, because of their contribution to the structure of biological membranes, are part of the fundamental group of nutrients for humans. Lipids can store energy and act as key signaling molecules. Fatty acids are the components of lipids. Fatty acids (FA) can be divided into Saturated (SFAs) having zero double bonds, monosaturated (MUFAs) having one double bond, and PUFA having 2–6 double bonds. At present, essential fatty acids are treated as functional foods and nutraceuticals having various advantages for human health that include the decrease in the risk of cardiovascular diseases, diabetic illness, cancer, osteoporosis, neurocognitive function, and diseases related to autoimmune issues.

Lecithin, which is also a lipid and a sticky fatty substance, has a composition of phospholipid mixtures along with neutral lipids, a small portion of glycerides, and other suspended matters. The use of lecithin is wide because of its emulsifying properties. It is used in nutraceutical sectors, like lecithin nano-vesicles, as supplementary food. In general, marine lecithin is extracted and characterized from the residues of viscera of squid (*Todarodes pacificus*) with the help of a supercritical carbon dioxide (SC-CO_2_) extraction process. The two significant phospholipids of lecithin from squid viscera are phosphatidylcholine and phosphatidylethanolamine (94). It was found that there are some differences in the compositions of the phospholipids extracted from squid viscera due to their different nature of food intake, habitat, isolation processes, or fishing seasons. Some studies, however, showed an almost opposite composition of these squid viscera phospholipids (95).

When the Australian lobster (*Jasus edwardsii*) was used to extract the lipids from its liver using SC-CO_2_, it was found that it contains high concentrations of PUFA and very low portions of impurities like arsenic, cadmium, lead, and mercury (96). This suggests that the lipid derived from the lobsters could be used in preventing, as well as in treating, various disorders and diseases like asthma problems, diabetic symptoms, coronary heart disease, and rheumatoid arthritis among others. The skin of the Indian mackerel (*Rastrelliger kanagurta*) and by-products like the shell, head, and tail of the Northern shrimp (*Pandalus borealis*) are also useful to obtain the deep red oily omega-3 PUFAs like EPA and DHA (97). The maximum recovery of PUFAs was seen in the oil isolated from Indian mackerel. Research suggested that the highest recovery of PUFAs, like omega-6, 3, and DHA from the by-products of fishes such as heads of the long-tailed tuna (*Thunnus tonggol*), is possible when ethanol is used as a co-solvent of SC-CO_2_ (98).

Fascinatingly, the oils isolated from different types of salmon discards (trimmed muscles, belly parts, skin, and frame bone) *via* various techniques like SC- CO_2_, hexane extraction, and pressed oil did not demonstrate any modifications in the composition of fatty acid. Interestingly, oils extracted with SC-CO_2_ have higher antioxidant characteristics than those extracted using hexane, suggesting a substantial difference in free radical scavenging activity (99).

For example, Lyprinol^®^- a commercialized product, which is used in the market to minimize the inflammatory processes caused due to arthritis, is a lipid portion of the freeze-dried isolate from the farmed green-lipped mussel *Perna canaliculus* (100).

Regarding the lipids obtained from sources of wastes, it is critical to emphasize the significance of squalene, a bioactive compound. Squalene is a terpenoid lipid that is produced partially through endogenous biosynthesis of cholesterol and partly by dietary sources, particularly in communities that consume a lot of shark liver, olive oil, wheat germ, and olives rice bran. To ease skin irritations and/or tumors, squalene is wonderful since it possesses antioxidant and anti-cancer qualities, in addition to being a good emollient and skin moisturizer.

Vitamin E is a lipid-soluble antioxidant, which protects cell membranes from lipid peroxidation and is found in both plants and animals. The four homologous pairs (β-, α-, δ-, and γ- tocotrienols, and tocopherols) are described, while the most active is the α form. Tocopherols were originally extracted from salmon eggs, then, from organs and tissues of a variety of fishes and make them a significant source of such advantageous molecules.

### Carotenoids

Discards from the processing of crabs, salmons, and shrimps might be a significant source of carotenoids (like astaxanthin). On the one hand, the intake of carotenoids through supplements or diet should be carefully examined because humans lack particular biosynthetic pathways. Carotenoids, on the other hand, are intriguing biomacromolecules that can help to reduce the negative effects of oxidative stress. A relationship was discovered between increased food consumption, tissue carotenoid concentrations, and a decreased risk of chronic illnesses. Furthermore, astaxanthin’s antioxidant activity controls biological activities associated with lipid peroxidation, which can help prevent chronic illnesses including macular degeneration, cardiovascular disease, and cancer.

In the instance of astaxanthin, an oral treatment of 1 mg/kg/day for 14 days decreased hepatic metastasis in rats, implying that it plays an essential role in improving the immune response by inhibition of lipid peroxidation caused due to stress (101). With the increasing concentration of extract, astaxanthin and its esters showed a substantial antioxidant effect. Moreover, researchers have found that astaxanthin has an anti-proliferative impact on carcinoma cells in the larynx of humans (Hep 2 cells). Encapsulation in beads made from alginate and chitosan was explored to increase the stability of astaxanthin derived from shells of shrimp (*Litopenaeus vannamei*), allowing it to be used as a functional component (102, 103).

Fucoxanthin, a carotenoid derived from the macroalgae *Undaria pinnatifida*, could also be utilized to combat eutrophication and promote aquaculture’s long-term viability. Because of the diverse locations and months of collection, it was less as compared to the fucoxanthin concentration seen in Japanese consumer wakame products.

Due to the loss of fucoxanthin and phenols while processing, fucoxanthin isolated from fresh samples had a stronger antioxidant action as compared to the commercial algae (104). Likewise, fucoxanthin isolated from the *Sargassum wightii* (Greville) species demonstrated an in *vitro* antioxidant activity and suppression of ACE, suggesting that it might be used as a dietary additive to treat hypertension (105).

### Polysaccharides

The major sources of chitin in the aquatic environment like the sea are shrimp, crustaceans, and crabs. Chitosan is the most significant chitin derivative, which is made by partially deacetylating chitin by enzymatic hydrolysis or under alkaline conditions under the influence of a chitin deacetylase (106, 107). In various research, it has been shown that chitosan, along with its derivatives, exhibits anti-microbial, antioxidant, and anti-viral properties (108). Chitosan, chitin, and their derivatives can serve as the inhibitors of ACE, which is an enzyme linked to hypertension. Chemical methods employed to extract chitin from aquatic wastes (crabs and shells of shrimps) provided deproteinization and demineralization by using strong bases or acids. Enzymatic processes make use of enzymes, such as proteases, or through fermentation by microbes.

In many studies, it is revealed that the chitosan and chitin are present in a variety of marine organisms, such as in the *Rapanavenosa* (snail) eggs, *Eriphiaverrucosa* (marine crab) shell, or in the *Lutjanus* spp. (Red snapper fish) scales available for use in agriculture, biotechnological processes, and in industries. In addition to that, it was also found that the chiton shells are more enriched with chitosan and chitin compared to the commercially available products (109). The capacity of *Sepia prashadi* cuttlebone to store these compounds is comparable to other sources like shells of a crab. Sulfated polysaccharides, recovered by water and enzymatic extraction from the macroalgae Gracilaria debilis and Gracilaria caudata, respectively, demonstrated to have antioxidant properties in a concentration-dependent way (110). The overall antioxidant capacity of these polysaccharides was assessed *in vitro* by ferrous ion chelating potential, and *in vivo* through the use of an oxidative stress rat model produced by 2,2′-azobis (2-methylpropionamidine) dihydrochloride (ABAP).

Glycosaminoglycans [(GAGs) for example: sulfate, chondroitin, hyaluronic acid, and dermatan sulfate], a complex of carbohydrate, are another type of polysaccharide with remarkable bioactivities such as anti-metastatic, antiviral, anticoagulant properties, and anti-inflammatory, as well as a lot of possibilities in the tissue engineering field. The volume and distribution of sulfate groups throughout the disaccharide chain determine the medicinal effects. The volume and distribution of sulfate groups throughout the disaccharide chain determine the medicinal effects. The GAGs have been isolated from various marine creatures, including sea cucumbers, sea urchins, shrimps, and sea squirts that may constitute seafood discard from fisheries, aquaculture, and industrial activities. GAGs derived from the mussel *P. canaliculus* have been discovered to be effective as an anti-arthritic substance. The research was done on Biolane™, a commercial product that contains GAGs, as well as matrix metalloprotease (MMP’s), a group of enzymes required for proper tissue re-modeling. The major findings demonstrated that this marine-derived composition has a wide variety of therapeutic properties, including suppression of pro-inflammatory cyclooxygenase-2 (COX-2), prostaglandins (PGEs), fibrinolytic activity, and anti-platelet aggregation (111). Clinical trials have shown that a cold isolate from the same species of mussel, which is excessively high in glycosaminoglycans, can alleviate joint discomfort and improve joint mobility. Thr GlycOmega-PLUS™ was the brand name given to it.

Alginate is a polymer that is abundant in calcium, sodium, strontium, magnesium, and barium ions and is found in the intercellular matrix in brown algae. Alginate is frequently utilized in industry for its water-retention capability, viscosifier, gelling, and stabilizing qualities. Alginate might have numerous applications in the nutraceutical sector, in addition to its usage as a printing paste in the textile industry. Alginate, extracted from the alga *Sargassum angustifolium* using several procedures (acid, water, cellulase, and alkalase), demonstrated antioxidant action in a dose-dependent way (112). Fucoidan, a category of sulfated heteropolysaccharide compounds enriched with fucose found in cell walls of brown seaweed and certain aquatic invertebrates, was used as dietary fiber in several Asian nations (113). The fucoidan structure differs depending on the species, but it normally comprises sulfate and L-fucose, along with minor amounts of D- mannose, D-galactose, D-xylose, and uronic acid. These fucoidans showed anti-inflammatory, antioxidant, anti-tumor, anti-allergic, anti-coagulant, anti-obesity, anti-hepatopathy, anti-viral, anti-renalpathy, and anti-uropathy impacts among other biological activities.

For example, fucoidan isolated from the brown-colored algae *Sargassum polycystin* demonstrated antioxidant property at 1,000 μg/mL and showed anti-proliferative action with an IC50 of 50 g/mL against the breast cancer cell-line in humans (114). Furthermore, fucoidan from *Sargassum wightii* was shown to control postprandial hyperglycemia among patients suffering from diabetes by functioning as an inhibitor of α- D- glucosidase in a dose-dependent way (115).

### Phenols

The nutraceutical benefits ascribed to phenolic compounds as part of both human and animal diets are nearly infinite, including preventive effects against neurodegeneration, cardiovascular illness, and cancer. Despite the large concentration of polysaccharides on the macroalgae matrix, extraction and characterization of macroalgae phenolic compounds, notably phlorotannins, have been problematic owing to their specialized biological effects like anti-proliferative, antioxidant, or anti-diabetic. For example, a 2,5-dihydroxybenzoic acid extracted from the macroalgae *Undari pinnatifida* and *Laminaria digitata* demonstrated a good inhibitory effect against α-amylase, while an extract enriched with polyphenols extracted from *Sargassum vachellianum* seaweed exhibited a significant free radical scavenging activity, antimicrobial effects and absorbed UV A and UV B rays effectively (116). Another aquatic polyphenol called Dieckol extracted from the Ecklonia cava brown alga, on the other hand, demonstrated sleep increasing benefits by boosting non-rapid eye movement sleep and lowering alertness over the sleep hours. All these findings suggested that, unlike current hypnotics, Dieckol might be utilized as a viable herbal sleeping aid with little negative impact (117). Because they are utilized in eutrophication control and intensive aquaculture, these macroalgae represent a significant source of discards.

## Microbial Valorization of Sea-Food Discards

The process of the involvement of various biological components in the mechanism of detaching various food components from their matrices is known as bioconversion. This process is mainly performed in the presence of enzymes and microorganisms. Both processes are environmentally friendly, safe, and cost-effective. Various microbial fermentation techniques help in the production of various types of hydrolytic enzymes that help in the bioconversion of various wastes (3). The mechanism involving microbes in bioconversion is known as fermentation, which is an environmentally friendly and energy-saving process. This mechanism utilizes various types of living microorganisms like fungi, bacteria, or microalgae in the conversion of various raw materials to utilizable products. The use of lactic acid bacteria (LAB) has been one of the most common processes in the development of fermented fishery products (118). The efficiency involved in the process of fermentation is dependent on the initial size of the inoculum, initial pH, and the pH that is attained during the time of fermentation. The mechanism of bioremediation with the involvement of various organisms is considered to be a cost-effective process. This mechanism provides an alternative way to exploit and isolate compounds those are possessing biotechnological importance (119). The microorganisms that are associated with this process can be aerobic, anaerobic, or facultative. Microbes possess the ability to perform solid-state, liquid-state, or submerged state fermentation (7). Table 2 describes the valorization of sea discards by microbes.

**TABLE 2:** Valorization of sea discards by microbes.

Discards of Seafood	Organisms/enzymes	Product formed	References
Powder of shrimp shell	*Bacillus cereus*	Helps in the production of DNA protective compounds and also helps in the formation of reducing sugars	(120)
Waste of Shrimp	*Rhizopus oligosporus* and *Lactobacillus brevis*	Helps in the formation of chitin	(121)
Various fish discards	Various groups of lactic acid bacteria	Helps in the formation of peptones	(122)
Condensate of Tuna	*Lactobacillus futsaii* and *Candida rugosa*	Helps in the formation of glutamic acid.	(123)
The bone of Grass fish	*Lactobacillus mesenteroides*	Use of calcium supplement	(124)
Viscera of water fish	*Pediococcus acidilactici*	Helps in the development of oil	(125)
Tuna waste	*Bacillus licheniformis Lactobacillus plantarum;*	Helps in the development of aqua feed	(126)
Solid waste of aquaculture	Bioconversion mediated by aerobic microbial species	Helps in the formation of liquid wastes	(127)
Head of shrimp	*Bacillus licheniformis*	Helps in the formation of antioxidants	(128)
Waste of shrimp	*Lactobacillus acidophilus*	Helps in the formation of chitin and protein hydrolysate	(129)
Shell associated waste	Symbiotic group of Lactic acid bacteria	Helps in the formation of carotenoids	(130)
Shells of crab	*S. marcescens* and *L. paracasei*	Helps in the formation of chitin	(30)
Waste of fish	Mixed consortia of microorganisms	Helps in the formation of liquid fertilizers	(3)
Shells of Shrimp	Pseudomonas aeruginosa	Helps in the formation of chitin	(131)

## Conclusion and Future Perspectives

This review focuses on how the discards from seafood can be sustainably and environmentally friendly used for developing nutraceuticals or functional foods for the health benefits of the humans. All the reports, so far, suggested that seafood discards comprise of a wide variety of bioactive compounds and biomacromolecules that can be used for improving human health and wellness.

During previous times, fishing, hunting, and gathering were the three basic methods for continual food supply. In modern era, humans still rely on the seafoods as one of the major components for consumption. Because oceans cover over 70% of the surface of the Earth, their tremendous biodiversity makes them a good place to look for raw materials like bioactive and natural compounds. Marine organisms are becoming more important as the world’s human population grows, not only as a source of food with high- quality, but also as a source of diverse substances for the pharmaceutical, cosmetics, and nutraceutical sectors. In fact, various biomacromolecules with therapeutic qualities such as anti-microbial, antioxidant, anti-obesity, anti-proliferative, anti-fibrotic, anti- Alzheimer, sleep-enhancing, neuroprotection, wound healing, lowering of lipids, and protection of skin have been identified from the sea. The exploitation of aquatic resources in a sustainable manner to meet the expanding human population’s food needs places a significant strain on the planet’s natural resources. As a result, more logical use of the limited natural resources is required. Because marine goods, particularly fishery discards, are extensively available and can cure or prevent a variety of ailments, it is critical to make functional diets from them. Inefficient utilization of aquatic raw materials and widespread utilization of non-selective fishing gears result in the loss of up to 50% marine captures, which are discarded in the sea, as well as up to 80% seafood raw material, which is not processed and lost. Discards released during industrial seafood processing can be correctly handled to yield raw resources. For achieving sustainable production with a minimal environmental imprint, this management demands a green revolution in industrial processing, along with the implementation of traditional processing technologies with eco-friendly and cheaper ones. Furthermore, seafood waste is considered environmentally harmful and can pose a severe trash management challenge. We presented data showing the possibility to efficiently reuse wastes and by-products from fisheries and aquaculture that would, otherwise, go to trash instead of being used to make nutraceuticals of high- quality for human use.

The study of functional components and their use in nutraceuticals is a rapidly expanding topic. Fish bycatch and seafood industrial discards are rich in biologically active peptides, polyunsaturated lipids, polysaccharides, polyphenols, carotenoids, collagen, minerals, saponins, gelatin, phytosterols, and phycobiliproteins to name a few. Apart from the low costs, these raw materials are easily and abundantly available, increased rates of recovery, intriguing functional qualities of the isolated compounds, and the aforementioned possible uses are the main benefits of extracting waste-derived nutraceuticals.

Furthermore, the use of waste chemicals in integrative aquaculture brings up new possibilities. For example, edible seaweeds could be used in photo-depuration procedures for multispecies cultivation systems, allowing species to be sold as food or food supplements when the cycle of production is completed. Certainly, the exploitation of seafood discards in nutraceuticals has resulted in a variety of solutions to common problems, including recycling, improving industry profitability, reducing human foot printing, and assuring the sustainability of aquatic resources.

Nevertheless, several factors must be considered, such as the linkages between processing methods and final product functionality. More research is needed to determine the optimum protocols for ensuring the stability of aquatic biomacromolecules during storage and processing, as well as the homogeneity of bioactive organic compounds based on natural variability within fishing areas, seasons, and production methods. These latter challenges continue to hinder the successful application of seafood discards for the food industries and more study is needed to overcome them and enable the successful manufacture of nutraceuticals that benefit human health.

## Author Contributions

MN, DL, AD, TS, SP, and SG: conception and design of the experiments. RR, TS, SP, and HB: writing–original draft preparation. MN, DL, AD, TS, SP, SJ, AM, and HE: formatting and editing according to journal guidelines. MN, DL, AD, TS, SP, RR, SG, HB, and HE: writing–review and editing. All authors have read and agreed to the published version of the manuscript.

## Conflict of Interest

SP is employed by NatNov Bioscience Private Ltd. The remaining authors declare that the research was conducted in the absence of any commercial or financial relationships that could be construed as a potential conflict of interest.

## Publisher’s Note

All claims expressed in this article are solely those of the authors and do not necessarily represent those of their affiliated organizations, or those of the publisher, the editors and the reviewers. Any product that may be evaluated in this article, or claim that may be made by its manufacturer, is not guaranteed or endorsed by the publisher.
